# Using Absorbable Sutures for Traumatic Wound Closure to Avoid Additional Hospital Visits for Suture Removal During the COVID-19 Pandemic: A Randomized Controlled Trial

**DOI:** 10.7759/cureus.30012

**Published:** 2022-10-06

**Authors:** Ahmed F Alkandari, Diaa M Soliman, Sampath Madhyastha, Abrar A Alawadhi, Fatma A Alawadhi, Nawaf M Almotairi, Ous Alozairi

**Affiliations:** 1 Department of Surgery, Al-Adan Hospital, Ahmadi, KWT; 2 Department of Anatomy, Kuwait University, Jabriya, KWT

**Keywords:** prolene suture, vicryl suture, suture removal, social distancing, hospital load, covid-19 pandemic, wound infection, traumatic wounds, non-absorbable sutures, absorbable sutures

## Abstract

Introduction

The COVID-19 pandemic worldwide forced governments to undertake intervention measures to encourage social distancing. Meanwhile, traumatic skin lacerations require multiple hospital visits for dressing changing and suture removal since they are usually repaired with non-absorbable sutures. These visits can be avoided by using absorbable sutures instead. However, absorbable sutures carry the “potential” risk of wound infection. In the current study, our first objective was to determine the non-inferiority of absorbable sutures regarding infection rate after repairing traumatic wound lacerations in comparison to the conventional non-absorbable ones. Our second objective was to evaluate the superiority of absorbable sutures in regard to postoperative clinic visits for suture removal and wound dressing compared to the non-absorbable ones.

Methods

A sample of 471 patients with traumatic skin lacerations was analyzed during the COVID-19 pandemic in April 2020. In the control group, wounds were repaired using non-absorbable sutures, while rapid-onset absorbable sutures were used in the treatment group. By conducting a phone/video call follow-up after 21 days, several parameters regarding infection signs and clinic visits were compared between both groups.

Results

A significant decrease in total trauma patients (45.4%) and those who required suturing (51.2%) was observed in April 2020 compared to the same month of the previous four years (p = 0.001 (2016), p = 0.027 (2017), p = 0.027 (2018), and p = 0.001 (2019)). Regarding wound infection, no statistically significant difference (p = 0.623) was observed between the absorbable (3.2%) and non-absorbable (4.9%) groups. Using absorbable sutures resulted in significantly (p < 0.001) fewer postoperative hospital visits compared to using non-absorbable sutures (mean: one versus three visits).

Conclusion

Using absorbable sutures to repair traumatic wound lacerations is safe regarding wound healing and infection rates. They also reduce postoperative hospital visits since they are not intended to be removed. Therefore, they should be considered during a pandemic to reduce hospital visits for suture removal, which will subsequently enhance social distancing and relieve hospital load.

## Introduction

The COVID-19 pandemic around the world forced the Kuwait Government to undertake intervention measures to encourage social distancing [1]. Traumatic skin lacerations, on the other hand, require multiple hospital visits for dressing changings and suture removal since they are usually repaired with non-absorbable sutures [2]. These visits can be avoided by using absorbable sutures, which will enhance social distancing, preserve medical equipment, and relieve hospital load during a pandemic [3]. However, absorbable sutures carry the “potential” risk of wound infection. Many researchers challenged this, but no substantial agreement exists [4].

In an attempt to avoid additional appointments for suture removal, Santos et al. suggested using absorbable sutures to close the skin after neurosurgical procedures during the pandemic [5]. In addition, in a case series of three patients, Roybal et al. found that using different wound closure techniques was beneficial in dermatological surgeries on elderly patients [6]. Moreover, after interviewing 35 patients who underwent skin surgeries using absorbable sutures, most would prefer to have absorbable sutures again if they had surgery in the future [7].

However, no such recommendations were set for repairing traumatic skin lacerations in the emergency departments. Therefore, we aimed to investigate the advantages of using absorbable versus non-absorbable sutures for the closure of traumatic wound lacerations during the COVID-19 pandemic. The first and second objectives were to assess the infection rate and the number of postoperative clinic visits between two groups, respectively.

This article was previously presented as a meeting abstract at the 26th Health Sciences Center (HSC) Poster Conference on March 22-24, 2022. In addition, this article is accepted as a poster presentation in the Scientific Forum at the American College of Surgeons Clinical Congress on October 16-20, 2022.

## Materials and methods

Ethical approval

The approval of the current study was obtained by the chairman of the general surgery department and the director of Al-Adan Hospital under the Institutional Review Board (IRB reference number: 2061/2022). This study was registered with the Ministry of Health of Kuwait with registration number MOH/2022/07/552.

Study design

This proof-of-concept study consisted of a parallel-group design randomized controlled trial.

Population

This study was undertaken at Al-Adan Hospital, Kuwait, in April 2020. This hospital covers a large number of people and serves over 40,000 patients a month. Each day, an average of 120-160 trauma cases attend the Casualty Operation Theater (OT), and about 60 patients of them require suturing as will be discussed in the later sections.

Sampling

Simple random sampling was used in the current study. All patients who attended the Casualty OT with traumatic wound lacerations requiring sutures were recruited in the trial.

Participants

**Figure 1:**
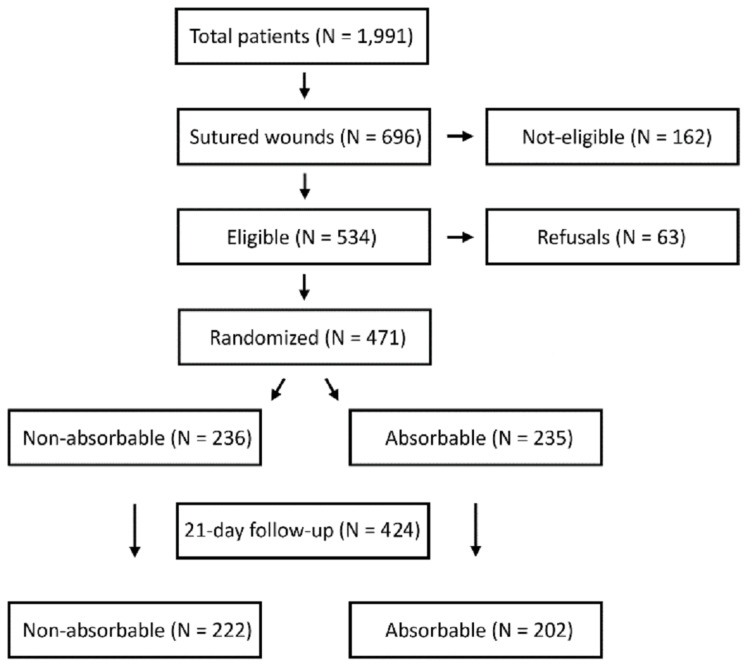
Selection of study participants

A total of 1,991 patients with trauma were seen in the Casualty Operation Theater in April 2020. Of these, 696 (35%) had lacerations that required suturing. Of the 696 patients, 534 (78%) met the inclusion criteria listed in Table 1 and were deemed eligible for the study. All participants who entered the sampling pool were provided with the objectives of the study and a brief study description. Sixty-three (12%) patients declined to participate. Of the remaining 471 patients, 236 were randomized to the non-absorbable group and 235 to the absorbable group. The rate of adherence to the study was 90.1%. A total of 222 (94%) patients in the non-absorbable group and 202 (86%) patients in the absorbable group were reached for the 21-day follow-up via a phone/video call, and statistical analyses were performed on this cohort (Figure 1).

**Table 1:** Criteria for inclusion in the trial

Patient-related criteria	Wound-related criteria
Healthy individual	Open traumatic wound
Ages between one and 60 years	More than 2 cm in length
Not on antibiotics	Within 24 hours duration
Not on anti-inflammatory medications	Located at areas of no tension (away from joint or bony prominence)

Randomization

Of the 534 patients eligible for the study, 471 patients were randomized into the two groups. Allocation concealment was ensured by using dice as a randomization method after the enrollment of the patients in the study. Odd and even numbers were used to allocate them to the non-absorbable and absorbable groups, respectively.

Intervention

To avoid technical variabilities, the 471 participants were divided into two groups where the wounds were repaired by a consistent surgeon using a simple interrupted technique. In the control group (n = 236), patients’ wounds were repaired using non-absorbable polypropylene sutures (Prolene, Ethicon Inc., Somerville, NJ, USA), whereas in the treatment group (n = 235), absorbable coated polyglactin 910 sutures (Vicryl Rapide, Ethicon Inc.) were used. In both groups, the wounds were cleaned with sodium chloride 0.09% solution prior to suturing. The suture thread size and the time for suture removal were determined according to the wounds’ location [2], as illustrated in Table 2. After suturing, povidone-iodine 10% w/v solution was applied to the wounds, followed by a sterile paraffin non-adherent gauze dressing. The wounds were then covered by different wound dressings, such as sterile adhesive wound dressing, non-woven fixation dressing tape, or dry dressing bandage according to their location.

**Table 2:** Guidelines for suture size and days of removal

Suture gauge	Suture removal day
Face: 6-0	Eyelids: 3 days
Rest of the head and neck: 5-0	Neck: 3-4 days
Trunk and upper limb: 4-0	Face: 5 days
Back and lower limb: 3-0	Scalp: 7-14 days
	Trunk and upper limb: 7 days
	Back and lower limb: 8-10 days

Wound care instructions

The patients were discharged with oral painkillers (paracetamol) and wound care instructions. No antibiotics were prescribed. In the non-absorbable group, the patients were instructed to visit the clinic after 7-10 days for suture removal. The patients were told that no further clinic visit is required for suture removal in the absorbable group. Additional clinic visits were recommended for both groups if any sign of infection appeared.

Data collection and follow-up

The contact phone number was taken for follow-up purposes. Age and gender were taken for demographic reasons. The time of injury and hospital arrival, the wound location, and the number of sutures were taken before and after the management. A total of 222 (94%) patients in the non-absorbable group and 202 (86%) patients in the absorbable group were reached for the 21-day follow-up. Several parameters regarding infection signs and clinic visits were taken by conducting a phone/video call.

Data analysis

Data were collected, coded, and entered into an IBM-compatible computer using the Statistical Package for the Social Sciences (SPSS) (version 27) statistical analysis software for Windows (IBM SPSS Statistics, Armonk, NY, USA). The entered data were checked for accuracy, then for normality using Kolmogorov-Smirnov and Shapiro-Wilk tests, and proved to be normally distributed. Qualitative variables were expressed as numbers and percentages, while quantitative variables were expressed as median, mean, and standard deviation (SD). The arithmetic mean was used as a measure of central tendency, while the SD was used as a measure of dispersion. Data analysis was done using the appropriate statistical significance test according to the objectives. A 5% level was chosen as a level of significance in all statistical significance tests.

Primary and secondary outcomes

The primary outcome was the assessment of the non-inferiority of absorbable sutures regarding infection rate after repairing traumatic wound lacerations in comparison to the conventional non-absorbable ones. The secondary outcome was the assessment of the superiority of absorbable sutures in regard to postoperative clinic visits for suture removal and wound dressing compared to the non-absorbable ones.

## Results

Number of trauma cases attending the Casualty OT

**Figure 2:**
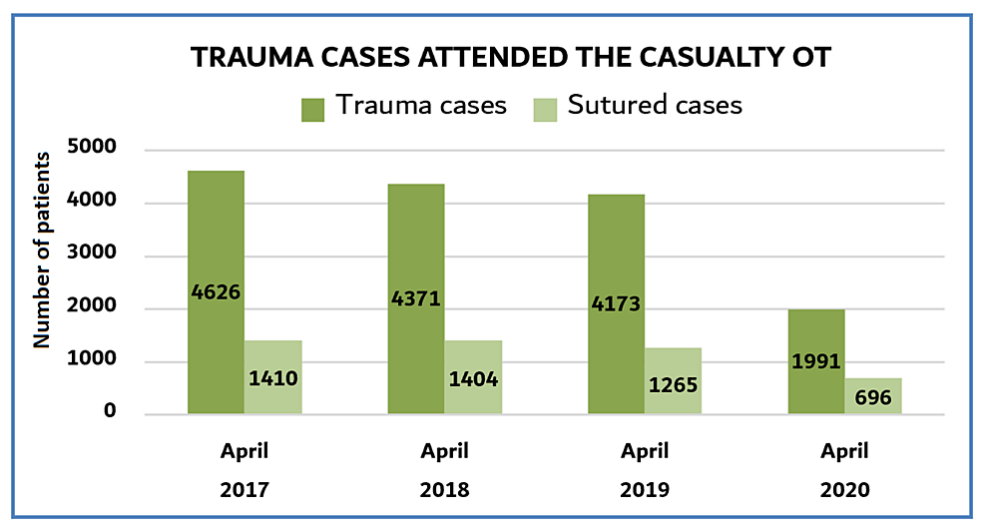
Trauma cases that attended the Casualty OT Due to the imposed curfew in the country and the fear of visiting healthcare centers during the pandemic, there was a significant decrease in the number of hospital visits compared to those in previous years.

The number of trauma cases attending the Casualty OT, including those that required suturing, decreased during the COVID-19 pandemic (Figure 2). We observed a significant decrease in total trauma patients (45.4%) and those who required suturing (51.2%) in April 2020 compared to the same month of the previous three years (p = 0.027 (2017), p = 0.027 (2018), and p = 0.001 (2019)).

Demographic data

The demographic characteristics of the two groups are summarized in Table 3. There were no significant differences in age, gender, wound duration, wound length, or the number of sutures in the two groups of patients who completed the entire study. The median age was 28 years (ranging from one to 60 years). The patients randomized to the absorbable group seemed to be slightly younger (median age: 23 years; interquartile range (IQR): 28) than those randomized to the non-absorbable group (median age: 31 years; IQR: 13). However, this age difference did not reach statistical significance.

**Table 3:** Demographics of the enrolled patients Data are presented as mean ± SD or n (%). SD: standard deviation; n: number

	21-day follow-up (N = 424)		
Groups	Non-absorbable (N = 222)	Absorbable (N = 202)	p-value
Age (year)	31.9 ± 4.2	23.4 ± 6.3	0.27
Gender (male/female)	153/69	135/67	0.75
Wound duration (hour)	1.8 ± 0.9	2.2 ± 0.6	0.58
Wound length (cm)	2.3 ± 1.8	3.1 ± 1.4	0.38
Number of sutures (%)	5 ± 1	4 ± 2	0.83

Wound characteristics

Table 4 summarizes wound complications concerning wound locations. Interestingly, wound infection occurred only in wounds located in the upper and lower limbs. Seven patients in the non-absorbable group developed wound infection; five had their injuries located in the upper limb and two in the lower limb. On the other hand, 10 patients in the absorbable group developed wound infection; seven had their wounds located in the upper limb and three in the lower limb.

**Table 4:** Wound locations and their relation to the number of sutures, infection, and suture sinus

Location	Number of sutures	Infection	Suture sinus
Scalp	84	0	0
Face	112	0	0
Neck	1	0	0
Trunk	2	0	0
Back	1	0	0
Upper limb	163	5 non-absorbable, 7 absorbable	0
Lower limb	61	2 non-absorbable, 3 absorbable	2 non-absorbable
Total	424	7 non-absorbable, 10 absorbable	2 non-absorbable

Outcomes

Wound Infection

**Figure 3:**
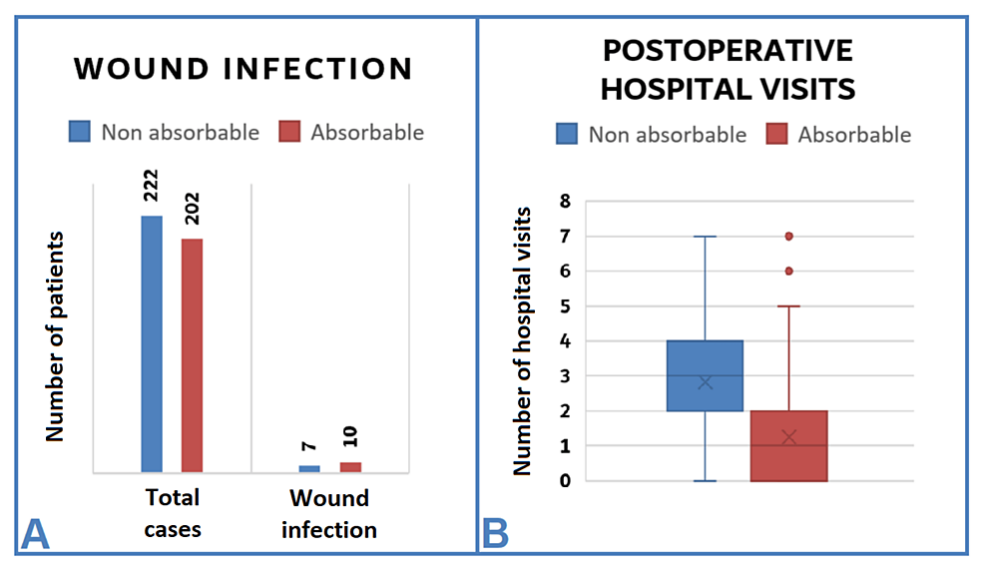
Wound infection and postoperative hospital visits (A) Wound infection: No statistically significant difference (p = 0.623) was observed between the two groups in terms of wound infection. (B) Postoperative hospital visits: Using absorbable sutures resulted in significantly (p = 0.001) fewer postoperative hospital visits compared to using non-absorbable sutures with an average ratio of 1:3.

No statistically significant difference (p = 0.623) was found between both groups regarding wound infection (Figure 3A).

Hospital Visits

Using absorbable sutures resulted in significantly (p < 0.001) fewer postoperative hospital visits. The average postoperative hospital visit ratio between the absorbable and non-absorbable groups was 1:3 (Figure 3B).

Suture Removal

**Figure 4:**
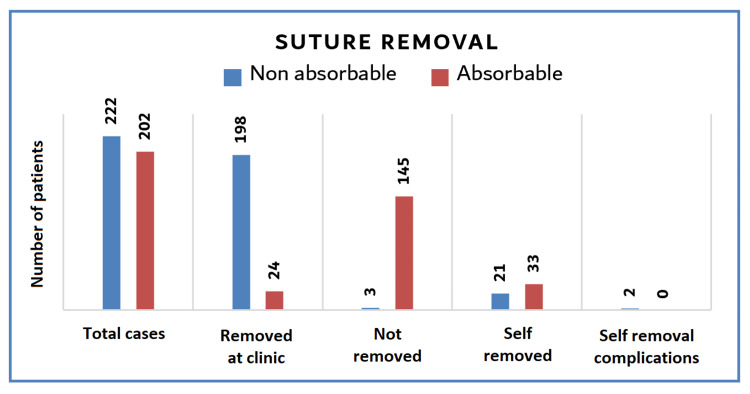
Suture removal The number of patients who removed the sutures at the clinic was significantly (p = 0.001) higher in the non-absorbable group. The number of patients who did not remove the sutures at all was significantly (p = 0.001) more in the absorbable group. Patients in both groups attempted self-removal of the sutures, although no significant difference exists (p = 0.52). Only two patients had self-removal-related complications in the non-absorbable group.

The number of patients who removed the sutures at the clinic was significantly (p < 0.001) more in the non-absorbable group. The number of patients who did not remove the sutures was significantly (p < 0.001) more in the absorbable group. Patients in both groups attempted self-removal of the sutures, although no significant difference exists (p = 0.52). Regarding self-removal complications, two patients had suture remnants in the non-absorbable group with subsequent skin sinus (Figure 4).

## Discussion

The impact of the COVID-19 pandemic on the healthcare system

During the COVID-19 pandemic, there was a significant impact on the availability of personal protective equipment, surgical masks, protective gowns, and other medical supplies [8]. There was a decrease in medical equipment as well as in the capacity of beds in hospital wards [9], things that forced the governments to limit hospital admission [9] and cease non-urgent surgeries [10,11]. The decreased number of trauma patients who attended the Casualty OT and those who required suturing during the study period (Figure 2) was influenced by the imposed curfew in the country and the fear of visiting the hospital during the pandemic [12].

The use of absorbable sutures during the pandemic

Wound Infection

The use of absorbable sutures did not result in a significantly increased risk of infection compared to the conventional non-absorbable ones (Figure 3A). This finding was similar to previous researchers who compared the outcomes of absorbable and non-absorbable sutures for repairing clean wounds in the face [13,14]. However, Tejani et al., in a randomized controlled trial on 115 patients with traumatic facial lacerations and other locations of cosmetic importance, found increased inflammation and scarring when using absorbable sutures compared to the non-absorbable ones [15]. This diversity may be due to the substantial variances in the inclusion criteria and the suture material used for skin closure. Therefore, our study used specific inclusion criteria listed in Table 1. Although there was no significant difference (p = 0.623) in wound infection between the groups in our study, wound infection only occurred in wounds located in the upper and lower limbs in both groups, as illustrated in Table 4. This finding may be because lacerations situated in the trunk and the extremities tend to heal more slowly and not as well as wounds on the face [16]. Thus, prophylactic antibiotics might be helpful for lacerations in areas with less circulation (upper and lower limbs) compared to areas with rich blood supply (scalp and face).

Hospital Visits

Using non-absorbable sutures has resulted in more hospital visits than using the absorbable ones (Figure 3B). The increased number of hospital visits in the non-absorbable group was due to dressing changes and suture removal or infection. The advantages of using absorbable sutures include the unnecessary need for follow-up care, decreased cost of care, and possibly decreased anxiety for children, as suture removal is unessential.

Suture Removal

**Figure 5:**
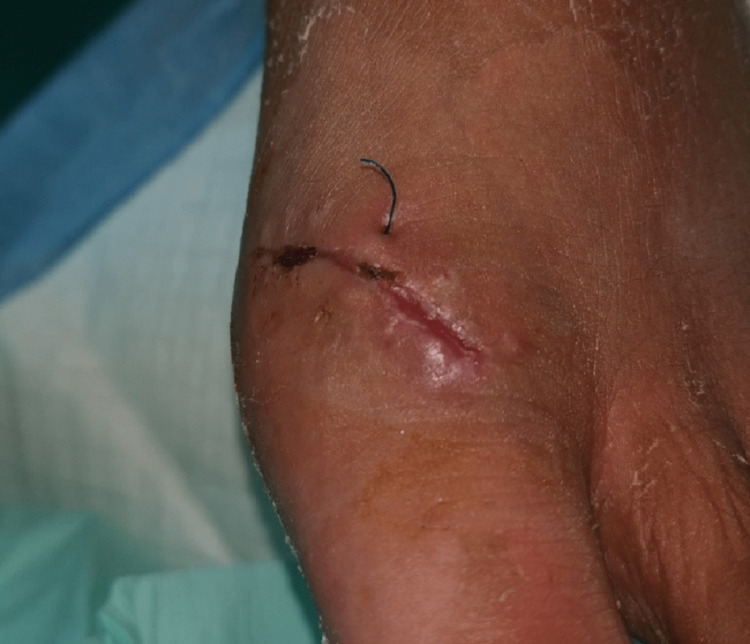
Suture remnant after inadequate self-removal of non-absorbable sutures Despite the instructions to remove the sutures at the clinic, the patient here removed them by himself, resulting in a buried knot under the skin. Upon questioning the reason behind his attempt, he stated that it was due to the fear of visiting the clinic during the pandemic.

Although the instructions of no clinic visits are required to remove the sutures, 24 patients in the absorbable group (11.8%) removed the sutures at the clinic due to either the delay in absorption or infection. Three patients in the non-absorbable group (1.3%) did not visit the clinic to remove the sutures due to the fear of coronavirus. Upon conducting the follow-up phone/video call after 21 days, they were directed to remove them at the clinic. Patients in both groups attempted to remove the sutures by themselves. In the non-absorbable group (n = 21) (9.4%), the reason for self-removal of sutures was the fear of visiting the clinic due to coronavirus. In the absorbable group (n = 33) (16.3%), the reason for self-removal of sutures was related to discomfort or delay in absorption. However, non-absorbable sutures can cause foreign body reactions and suture sinus if removed inadequately. Two patients in the non-absorbable group (n = 2) (0.09%) had suture remnants under the skin due to inadequate self-removal (Figure 4). A photograph of a suture remnant due to inadequate self-removal of sutures at home is shown in Figure 5. The complications of suture removal are well established [17]. Too early suture removal may cause wound dehiscence. In addition, late removal may lead to scar formation, foreign body reaction, or wound infection. Moreover, inadequate removal results in suture remnants under the skin that eventually cause suture sinus and its related consequences [18].

Limitations and recommendations

The first limitation is that our study was conducted only in one hospital in Kuwait and did not cover other hospitals. However, this hospital is one of the leading five governmental hospitals in Kuwait, as it covers approximately 600,000 people and treats over 40,000 patients a month [19,20]. Kuwait has a total population of 4.35 million as of April 8, 2022 [21]; therefore, Al-Adan Hospital covers around 15% of the total population in the country. The second limitation is that the assessment of wound infection was subjective to the patients by a phone/video call follow-up. Although this may influence our results, we preferred this method of data collection due to the circumstances of the COVID-19 pandemic. Besides, careful wound care instructions were provided to the patients after suturing, together with the symptoms and signs of wound infection. The third limitation is that the 21-day follow-up period may not be enough to show complications related to inadequate suture removal, such as skin sinus, which could result in a secondary skin infection. Future researchers may consider adding another follow-up session in return. Finally, the cosmetic result was not an objective of our present study; however, many authors, particularly plastic surgeons, found no significant differences in cosmetics between the two suture materials [22].

## Conclusions

Using absorbable sutures to repair traumatic wound lacerations is safe regarding wound healing and infection rates. They also reduce postoperative hospital visits since they are not intended to be removed. Therefore, they should be considered to reduce additional hospital visits, enhance social distancing, and relieve hospital load, especially during a pandemic. Moreover, using absorbable sutures could prevent unwanted complications of the non-removal or self-removal of non-absorbable sutures.
